# EIYMNVPV Motif is Essential for A1CF Nucleus Localization and A1CF (-8aa) Promotes Proliferation of MDA-MB-231 Cells via Up-Regulation of *IL-6*

**DOI:** 10.3390/ijms17060811

**Published:** 2016-05-25

**Authors:** Li Zhou, Jin Hao, Yue Yuan, Rui Peng, Honglian Wang, Dongsheng Ni, Yuping Gu, Liyuan Huang, Zhaomin Mao, Zhongshi Lyu, Yao Du, Zhicheng Liu, Yiman Li, Pan Ju, Yaoshui Long, Jianing Liu, Qin Zhou

**Affiliations:** 1 The Division of Molecular Nephrology and the Creative Training Center for Undergraduates, The M.O.E. Key Laboratory of Laboratory Medical Diagnostics, the College of Laboratory Medicine, Chongqing Medical University, Chongqing 400016, China; shmily525520@sina.com (L.Z.); lanyxiu@163.com (J.H.); yyokyy1126@hotmail.com (Y.Y.); pengrui911@foxmail.com (R.P.); cqmunds@163.com (D.N.); guyupinglittle@sina.com (Y.G.); lyhuang0603@sina.com (L.H.); mao1204086118@163.com (Z.M.); Zhongshilyu@163.com (Z.L.); duyao_steve@163.com (Y.D.); liuzhicheng323@163.com (Z.L.); 18715854036@sina.cn (Y.L.); 18883936591@163.com (P.J.); lys960110@sina.com (Y.L.); keithljn@sina.cn (J.L.); 2 Laboratory of Organ Fibrosis Prophylaxis and Treatment by Combine Traditional Chinese and Western Medicine, Research Center of Combine Traditional Chinese and Western Medicine, Affiliated Traditional Medicine Hospital of Sichuan Medical University, Luzhou 646000, China; hackie_wang@126.com

**Keywords:** Apobec-1 complementation factor (A1CF), EIYMNVPV motif, Interleukin-6 (*IL-6*), nucleus localization, proliferation, MDA-MB-231 cells

## Abstract

Apobec-1 complementation factor (A1CF) is a heterogeneous nuclear ribonuceloprotein (hnRNP) and mediates apolipoprotein-B mRNA editing. A1CF can promote the regeneration of the liver by post-transcriptionally stabilizing Interleukin-6 (*IL-6*) mRNA. It also contains two transcriptional variants-A1CF64 and A1CF65, distinguished by the appearance of a 24-nucleotide motif which contributes to the corresponding eight-amino acid motif of EIYMNVPV. For the first time, we demonstrated that the EIYMNVPV motif was essential for A1CF nucleus localization, A1CF deficient of the EIYMNVPV motif, A1CF (-8aa) showed cytoplasm distribution. More importantly, we found that A1CF (-8aa), but not its full-length counterpart, can promote proliferation of MDA-MB-231 cells accompanied with increased level of *IL-6* mRNA. Furthermore, silencing of *IL-6* attenuated A1CF (-8aa)-induced proliferation in MDA-MB-231 cells. In conclusion, notably, these findings suggest that A1CF (-8aa) promoted proliferation of MDA-MB-231 cells *in vitro* viewing *IL-6* as a target. Thus, the EIYMNVPV motif could be developed as a potential target for basal-like breast cancer therapy.

## 1. Introduction

Apobec-1 complementation factor (A1CF), also known as ACF, is the RNA binding subunit of a minimal core protein complex for apolipoprotein-B (apoB) mRNA edition [1,2,3]. Coordinated with APOBEC-1, A1CF regulates site-specific C to U edition of apoB mRNA, leadings to the truncated isoform apoB48, rather than apoB100. During this process, A1CF recognizes an AU-rich motif (mooring sequences) on apoB mRNA and guides APOBEC-1, the cytosine deaminase, to localize to the right site to edit [1,4,5,6,7,8]. However, the binding of A1CF to RNA may not be restricted to mooring sequence. It was reported that A1CF can promote liver regeneration after hepatectomy by binding *IL-6* mRNA at the AU-rich region of 3′-UTR, which shows no typical mooring sequence, to stabilize its expression [9]. Furthermore, germ-line deletion of *A1CF* directly resulted in embryonic lethality due to the defect of early embryo implant [10]. Knockdown of *A1CF* decreased proliferation in rat hepatoma cells [11]. In addition, A1CF contains three non-identical RNA recognition motifs (RRM) in its N-terminus and a unique C-terminal auxiliary domain, which are required for complementing activity, RNA binding and apoB mRNA editing [12]. Dance *et al.* demonstrated that the A1CF exon 11 is alternatively spliced to include or exclude 24 nucleotides at exon 12, leading to two variants, ACF65 and ACF64. Both variants are equivalent for apoB mRNA edition in cells [13]. In order to highlight the structure characters of the two variants, we designate the one excluding EIYMNVPV motif (A1CF64) as A1CF (-8aa), and the other including the EIYMNVPV motif (A1CF65) as A1CF. Fossat *et al.* reported that RBM47 interacted with APOBEC1 and A1CF and also took part in the C to U RNA edition. Furthermore, RBM47 can replace A1CF to complement APOBEC1’s RNA edition *in vitro* [14]. Given the important role of RBM47 in progression of breast cancer, and similar structure, function between RBM47 and A1CF [15], we hypothesize that A1CF (-8aa) or A1CF may also play a biological role in breast cancer.

Breast cancer is one of the most common malignancies in women with multifactorial etiology. In recent years, the treatments of breast cancer focus on the identification of cytokines as prognostic factors, including several interleukins (IL-1, IL-6, IL-10), transforming growth factor-β (TGF-β), tumor necrosis factor-a (TNF-a) and so on [16]. Among them, IL-6 is a secreted multifunctional cytokine and mainly functions in the host immune defense and the modulation of growth and differentiation in various malignancies [17,18,19,20,21,22]. It has been reported that IL-6 is involved in stimulating proliferation in many cancers, such as colorectal cancer, prostate cancer, intestinal disease, and so forth [23,24,25]. Meanwhile, recent studies have identified IL-6 as a prognostic factor in the treatment of breast cancer. There existed a reduction in proliferation following *IL-6* knockdown on basal-like breast cancer cells including MDA-MB-231 and Hs578T cells [21,26]. Most importantly, it was confirmed that A1CF could bind to an unstable AU-rich region in *IL-6* at its 3′-UTR to functionally regulate *IL-6* mRNA stability at posttranscriptional level [9,11]. However, whether *IL-6* is a downstream effector of A1CF (-8aa) or A1CF in breast cancer progression remained unclear.

In this study, we demonstrated that the EIYMNVPV motif is essential for the nuclear localization of A1CF in MDCK cells and MDA-MB-231 cells. A1CF (-8aa), but not A1CF, could promote the proliferation of MDA-MB-231cells via upregulating *IL-6*, However, A1CF (-8aa)-mediated proliferation can be inhibited by silence of *IL-6*.

## 2. Results

### 2.1. Bioinformatics Analysis of A1CF

**Figure 1:**
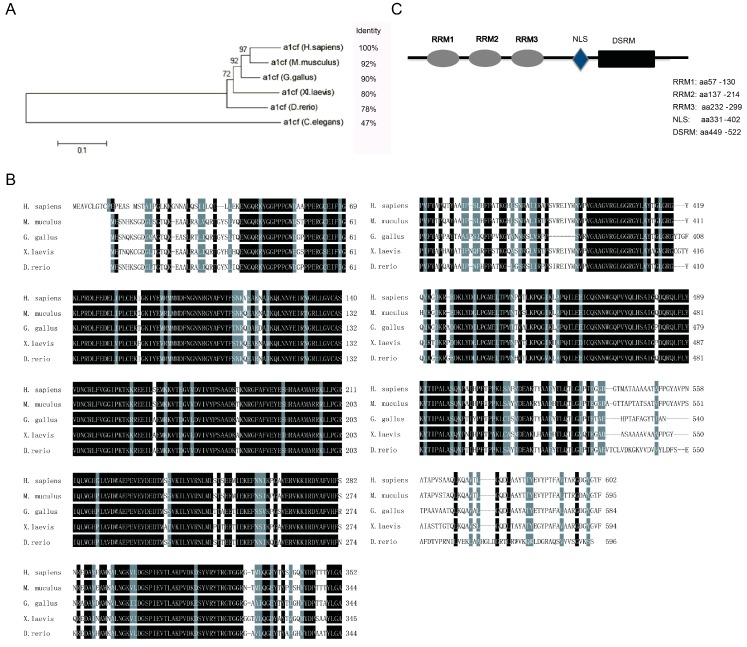
A1CF is conservative on evolution via bioinformatics analysis. (**A**) High conservation of A1CF across species in the schematic diagram of phylogenetic analysis. The similarity of amino acids sequences among species was shown as percentage; (**B**) Amino acids sequences alignment of A1CF among *Homo sapiens*, *Mus musculus*, *Gallus gallus*, *Xenopus laevis*, and *Denio rerio*. The identical amino acids were shaded in black and the similar amino acids were in grey; and (**C**) the predicted protein structure of *mus-A1CF* on the net. It contains three non-identical RRM on the N terminus, a nucleus localization signal, and a dsRBM on its C terminus, RRM, RNA recognition motif. ASN, A1CF specific nuclear signal. DSRM, double-strand RNA binding motif.

To show the characteristic of A1CF, we respectively analyzed the evolution tree and the conservation of A1CF among mammal species using MEGA5 software (http://www.megasoftware.net/), clustalW multiple sequence alignment software, and NCBI blast. The DNA sequences similarity rates among species had been shown on the right side of the evolution tree mainly ranging from 78% to 92% in relation to *Homo sapiens*, except that *Caenohabditis elegans* displayed the similarity of 47% (Figure 1A). Furthermore, the transcript variants of A1CF encoding the longest protein sequences were employed to do a comparative analysis of amino acids based on the DNAssist software among *Homo sapiens*, *Mus musculus*, *Gallus gallus*, *Xenopus laevis*, and *Danio rerio*. As descripted in Figure 1B, the results of alignment revealed a high conservation among them. Moreover, functional motif analysis prediction suggested that mus-A1CF contains three non-identical RNA recognition motifs (RRM) on the N terminus, a double-strand RNA binding motif (dsRBM) on C terminus, and a nucleus localization signal (Figure 1C), which is in accordance with the results in *Homo sapiens* reported by Valerie Blanc *et al.* [27]. All of the results further indicate that A1CF is highly conservative.

### 2.2. The Deletion of the EIYMNVPV Motif by Alternative Splicing is Conserved Across Species

**Figure 2:**
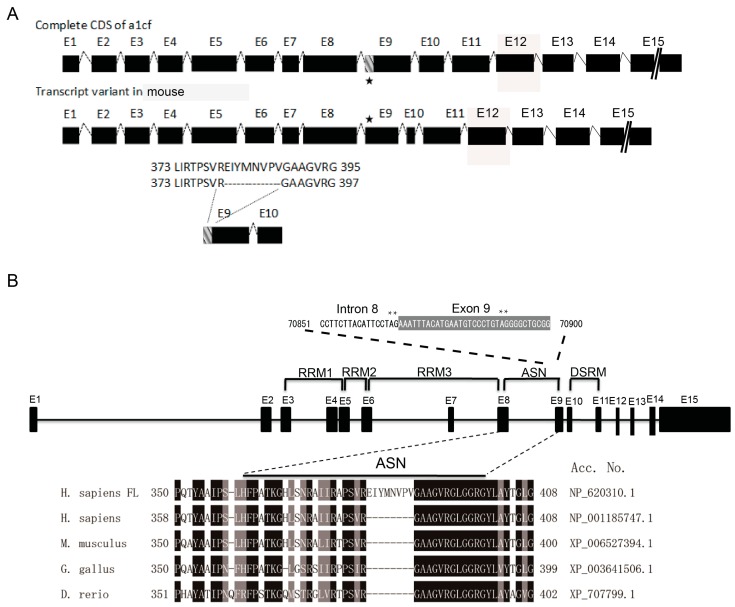
The conservation analysis of the EIYMNVPV motif deficiency (**A**) exon pattern of mouse *a1cf* transcript and its alternatively-spliced variant *A1CF (-8aa)*.An alternative splice in exon 9 results in a deletion of the EIYMNVPV motif. As the pentagram shows, this alternative splice takes place in the 5’ terminus of exon 9; and (**B**) the conservation of the EIYMNVPV motif deficiency among *H. sapiens*, *M. musculus*, *G. gallus*, and *D. rerio*. Human full length A1CF (FL) was used for comparison with A1CF (-8aa) from human, mouse, chicken, and zebrafish respectively, the asterisks represent an alternative splice acceptor site in the anterior region of exon 9. The identical amino acids were shaded in black and the similar amino acids were in grey.

Previous study has indicated that A1CF is alternatively spliced to include or exclude a 24-nucleotide motif in humans [13]. For mouse A1CF, this alternative splice takes place in the 5′ terminus of exon 9 (Figure 2A). To figure out whether this kind of special splice event happens in other evolutionarily lower-ordered species, we compared the corresponding mRNA region of A1CF transcripts from *H. sapiens* (*Homo sapiens*), *M. musculus* (*Mus musculus*), *G. gallus* (*Gallus gallus*) and *D. rerio* (*Denio rerio*). As shown in Figure 2B, transcript deficient of EIYMNVPV (A1CF (-8aa)) can be found from chicken and zebrafish. Further analysis show that there exist an alternative splice acceptor site in the anterior region of exon 9 (asterisk in Figure 2B). In conclusion, these data demonstrate that the alternative splice event leading to the A1CF (-8aa) isoform is evolutionarily conserved, which implies its biological importance.

### 2.3. EIYMNVPV Motif is Essential for A1CF Nucleus Localization

**Figure 3:**
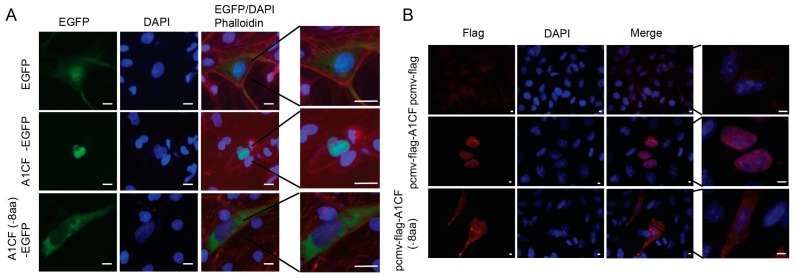
EIYMNVPV motif plays a vital role in A1CF nucleus localization. (**A**) Subcellular distribution of A1CF (-8aa)-EGFP and A1CF-EGFP in MDCK cells with the immunofluorescence technique. Green represents A1CF-EGFP, A1CF (-8aa)-EGFP, or EGFP (null vector); blue stains the nucleus; red is responsible for Phallodin; scale bar represents 10 μm, the scale bar of the sub Figure on the right: 10 μm; and (**B**) fluorescence immunostaining to show the subcellular distribution of Flag-A1CF (-8aa) and Flag-A1CF in MDA-MB-231 cells. Red is responsible for pcmv-flag-A1CF (-8aa), pcmv-flag-A1CF, and pcmv-flag, blue stains the nucleus; scale bar: 49 μm, the scale bar of the sub Figure on the right: 2 μm.

As is displayed in Figure 2B, the EIYMNVPV motif was located at the 381–388 aa region. However, it was reported that there existed a novel nuclear location signal, termed A1CF-specific nuclear signal (ASN), at the 331–402 aa region, which is indispensable for A1CF nucleus localization (Figure 2B) [27]. Therefore, it remains a concern that whether the absence of EIYMNVPV would impair the nuclear translocation ability of A1CF. To address this question, A1CF and A1CF (-8aa) were cloned, respectively, into proper vectors to express GFP-fused or Flag tag-labeled recombinant protein and the subcellular distribution was checked. As shown in Figure 3A, A1CF-EGFP fusion protein presented nuclear distribution in MDCK cells, while A1CF (-8aa)-EGFP was located in cytoplasm. However, the control protein, EGFP, demonstrated both cytoplasm and nuclear expression. To confirm the influence of the 8aa motif on A1CF subcellular distribution, the small sized flag tag was used to label A1CF protein to exclude the influence of EGFP on A1CF distribution. Similarly, in MDA-MB-231 cells, Flag-A1CF was located in nuclear. However, Flag-A1CF (-8aa) was restricted in cytoplasm (Figure 3B). Collectively, our findings demonstrated that the EIYMNVPV motif physiologically caused by alternative splicing played a critical role in A1CF nucleus localization.

### 2.4. A1CF (-8aa) Promotes Proliferation of MDA-MB-231 Cells

**Figure 4:**
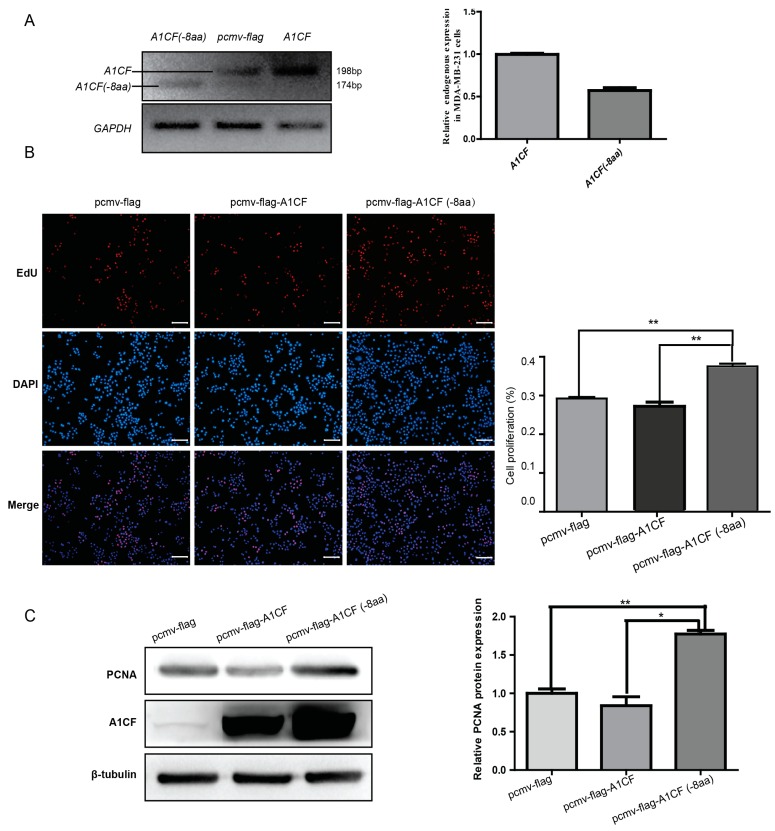
Ectopic expression of *A1CF (-8aa)* promotes the proliferation of MDA-MB-231 cells accompanied by upregulated *IL-6*. (**A**) RT-PCR to detect the endogenous and enforced expression of *A1CF (-8aa)* and *A1CF* expression in MDA-MB-231 cells, *GAPDH* was used as internal control. Cells transfected with pcmv-flag-A1CF and pcmv-flag-A1CF (-8aa) were used as positive control; (**B**) cell proliferation was checked by EdU incorporation assay. Proliferating cells were labeled with EdU (red), with nuclear stained DAPI (blue), scale bar: 100 μm. Statistic graph of the EdU staining was given on the right. Data were representative of three independent experiments, and error bars represent the standard deviation from the mean. ** *p* ≤ 0.01; and (**C**) MDA-MB-231 cells were transfected with pCMV-flag-A1CF or pCMV-flag-A1CF (-8aa) followed by Western blot to detect the expression of PCNA and A1CF. β-Tubulin was used as loading control. The statistic result was displayed on the right, and revealed that ectopic expression of *A1CF (-8aa)* resulted in a gain of proliferation marker (PCNA) compared with *A1CF* and negative control. * *p* ≤ 0.05; ** *p* ≤ 0.01.

To uncover the biological significance of A1CF (-8aa), we firstly investigate the expression pattern of *A1CF* and *A1CF (-8aa)* in MDA-MB-231 cells, a breast tumor cell line. Semi-quantitative PCR was used to analyze the relative expression abundance of *A1CF* and *A1CF (-8aa)*. As shown in Figure 4a, the predominant transcript isoform is A1cf, while weak expression of *A1CF (-8aa)* can be observed. Quantitative analysis indicated that A1CF showed twice the expression than that of *A1CF (-8aa)*. As is reported, RBM47 could replace A1CF, and regulate APOBEC1-mediating mRNA editing *in vitro* [14]. Additionally, it participates in the breast cancer progression via modulating mRNA stability [15]. To verify the possible correlation between A1CF (-8aa) and basal-like breast cancer cells, we respectively transfected MDA-MB-231 cells with *A1CF (-8aa)*, *A1CF* and control vector for 36 h. The 5-Ethynyl-20-deoxyuridine (EdU) and the expression of PCNA were examined to evaluate the proliferation effect of A1CF (-8aa) and A1CF on MDA-MB-231 cells. PCNA plays an important role in DNA synthesis, DNA repair, cell cycle progression and cell proliferation [28,29]. As Figure 4B displayed, the ratio of proliferative cells was increased in MDA-MB-231 cells treated with *A1CF (-8aa)*. In line with the EdU incorporation assay, PCNA, a marker for proliferation, showed increased expression in A1CF (-8aa)-transfected cells but not in A1CF-transfected cells as detected by Western blot (Figure 4C). All in all, the above findings indicated that A1CF (-8aa) rather than A1CF promote the proliferation of MDA-MB-231 cells.

### 2.5. IL-6 Was Involved in A1CF (-8aa)-Promoted Proliferation of MDA-MB-231 Cells

**Figure 5:**
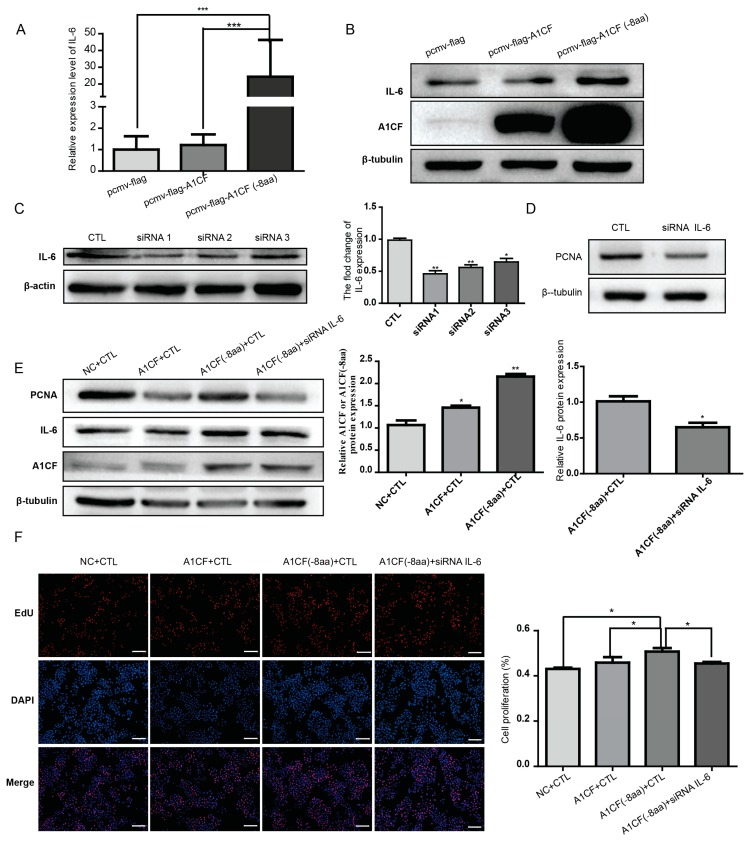
Silencing *IL-6* could attenuate the A1CF (-8aa)-induced proliferation of MDA-MB-231 cells. MDA-MB-231 cells were transfected with pCMV-flag-A1CF or pCMV-flag-A1CF (-8aa) or blank vector. Real-time PCR was performed to detect the RNA level of *IL-6* (**A**) and Western blot to detect the protein level of IL6 and A1CF (**B**); β-tubulin was used as loading control in (**B**); (**C**) the knockdown efficiency of *IL-6* siRNAs was detected by Western blot analysis. β-Actin was taken as loading control. Statistical results are shown on the right; (**D**) the expression of PCNA decreased after *IL-6* siRNA treatment as determined by Western blot. β-Tubulin was taken as internal control; (**E**) Western blot is used to show that the expression of *IL-6* and PCNA declined in cells co-transfected with *A1CF (-8aa)* and *IL-6* siRNA compared with controls. The right statistic data quantified the over-expression and silence efficiency of A1CF and *IL-6*, respectively; and (**F**) rescue assay of cell proliferation by *IL-6* siRNA as determined by EdU assay. Proliferating cells were labeled with EdU (red), and cell nuclei were stained with DAPI (blue), scale bar: 100 μm. The statistical graph is shown on the right. All of the data were representatives of three independent assays, and error bars were on behalf of the standard deviation from the mean. * *p* < 0.05; ** *p* < 0.01; *** *p* < 0.001.

It has been reported that A1CF can promote liver regeneration via *IL-6* by stabilizing *IL-6* mRNA [9]. Furthermore, IL-6 has been proved to play a vital role in breast cancer progression and prognosis. To unravel whether IL-6 mediates A1CF (-8aa)-promoted proliferation, we first checked whether A1CF (-8aa) could enhanced the expression of IL-6. As illustrated in Figure 5A and B, expression level of both mRNA and protein of IL-6 increased in MDA-MB-231 cells transfected with A1CF (-8aa), but not in cells transfected with A1CF. To further clarify the effect of *IL-6* in A1CF (-8aa)-mediated proliferation, RNA silence was used to knockdown IL-6. Three siRNA oligos were designed to screen the siRNA showing best expression silence of IL-6. As determined by western blot in Figure 5C, siRNA1 demonstrated best gene silence efficiency (more than 50% compared with control group). Thus, siRNA1 was used for downstream investigation. Transfection of IL-6 siRNA alone can downregulate the expression of PCNA in MDA-MB-231 cells (Figure 5D). Most importantly, knockdown of IL-6 can reverse the increased expression of PCNA stimulated by A1CF (-8aa) (Figure 5E). In accordance with decreased expression of PCNA, IL-6 knockdown can also rescue enhanced proliferating cell proportion induced by A1CF (-8aa) as determined by EdU incorporation assay (Figure 5F).

## 3. Discussion

A series of research findings from Nicholas’ group validated the vital role of A1CF for survival, as its deficiency will cause early embryo lethality in development, impaired liver regeneration after hepatectomy, and increased apoptosis of liver cancer cell [10,11]. A1CF is an hnRNP protein with shuttling ability between nuclear and cytoplasm. Yet now, the reported clear biological functions are direct participation in post-transcriptional RNA edition and mRNA stabilization. RNA edition is believed to be a nuclear event. However, A1CF stabilizes *IL-6* mRNA by binding to its AU-rich region should take place in cytoplasm. Blanc *et al.* first identified the nuclear translocation signal of A1CF. Although existence of the alternative transcript encoding A1CF (-8aa), also A1CF64, has been verified years ago, no biological significance has yet been found. Here, for the first time, we reported that the deleted 8aa, EIYMNVPV, was just localized in the internal region of A1CF nuclear translocation signal. Furthermore, functionally, we proved that absence of 8aa abrogated the nuclear localization ability of A1CF. Moreover, we further validated that A1CF (-8aa) but not A1CF contributed to the proliferation of breast cancer cells, thus again firstly uncovering the biological significance of A1CF (-8aa). Given the high-degree conservation of the alternative spliced transcript encoding A1CF (-8aa) across species, A1CF (-8aa)-regulated cell proliferation may have vital roles in varies of aspects. Still, more data are needed to verified the role of A1CF (-8aa) in cell proliferation and survival in other cell lines in addition to breast cancer cells.

Breast cancer is the one of the most common tumors in women with multifactorial etiology. And almost all of the existing strategies for breast cancer treatment are unsatisfactory, impressing the urgent and quite important mechanism research [16]. IL-6 and its downstream signal network (IL-6/IL-6R/gp130) are reported to play important roles in breast cancer. IL-6 was widely expressed in breast cancer and was involved in growth, metastasis of cancer cells, and renewal of cancer stem cells. Thus, IL-6 has already been one of the therapy targets and markers for disease prognosis [30]. In this study, we found that A1CF (-8aa) can promote proliferation of breast cancer cells mostly likely by stabilizing the mRNA of *IL-6*. These findings implicated a role of A1CF in *IL-6* signaling-mediated progress of breast cancer. However, before validation of the role of A1CF in breast cancer, more studies are required to address the prevalence of A1CF (-8aa) in breast cancer and whether blocking A1CF (-8aa) or the corresponding alternative splice event can help cancer therapy.

## 4. Materials and Methods

### 4.1. Plasmid Construction

For synthesis of *A1CF (-8aa)* and *A1CF in vitro*, the *A1CF (-8aa)* and *A1CF* fragment were cloned from cDNA by PCR with following primers: *pcmv-flag-A1CF* (F1: 5′-GGATCCACTAGTTCTATGGAATCAAATCACAAATCC-3′; R1: 5′-CACCCGGGATCCTCTGTTAGAAGGTTCCATATGCATCG-3′); *pEGFP-N1-A1CF* (F2: 5′-GATCTCGAGCTCAAGATGGAATCAAATCACAAATCC-3′; R2: 5′-TTCTGCAGTCGACGGGTTAGAAGGTTCCATATGCATCG-3′); Amplified fragment of *A1CF* was inserted into the pcmv-flag at the site of XbaI and pEGFP-N1 at the site of HindIII and XhoI, respectively. Then, plasmids of *pcmv-flag-A1CF (-8aa)* and *pEGFP-N1-A1CF (-8aa)* were separately amplified with the method of point mutation from mature plasmids of *pcmv-flag-A1CF* and *pEGFP-N1-A1CF*, with the primers: *A1CF (-8aa)* (F: 5′-AGGGACATTCATGTAAATTT CTCTGACAGA AGGGGTCCTG-3′; R: 5′-TACATGAATGTCCCTGTAG GGGCTGCGGG GGTGAGAGGA-3′). Finally, all the plasmids were verified by sequencing.

### 4.2. Cell Culture and Transfection

MDCK cells and MDA-MB-231 cells, as described previously [31,32], were respectively cultured in Dulbecco’s Modified Eagle Medium (DMEM) and RPMI-1640, supplemented with 10% fetal bovine serum (FBS, *vol*/*vol*, GIBCO,BRL Co., Ltd., Grand Island, NY, USA) and 0.1% penicillin/streptomycin (Invitrogen, Grand Island, NY, USA). All the cell lines were cultured at 37 °C with 5% CO_2_. For some experiments, cells was transiently transfected with overexpression plasmids of *A1CF (-8aa)*, *A1CF*, negative control, or *IL-6*-siRNA (50 nM) by using Lipofectamine 2000 reagent (Invitrogen, Grand Island, NY, USA) according to the manufacturer’s instructions. Then the cells were received at different time point, RNA or protein was extracted from those cells and stored at −80 °C for subsequent analyses. *IL-6*-siRNA duplexes sequences are as follows:

siRNA1:sense5′-GCAGACCCAAGAUACCCUA-3′, antisense 3′-UAGGGUAUCUUGGGUCUGC-5′;
siRNA2: sense5′-GGUCUGGCAUGUGACCAUU-3′, antisense3′-AAUGGUCACAUGCCAGACC-5′;
siRNA3:sense5′-GGGUGAUAGUGACAGUGAA-3′, antisense3′-UUCACUGUCACUAUCACCC-5′.

### 4.3. RNA Extraction, RT-PCR, Quantitative RT-PCR (qPCR)

Total RNA was extracted from the breast cancer cells with Trizol reagent (Ambion, Austin, TX, USA) on the basis of the manufacturer’s protocol. Adding 1 mL Trizol, and placing at 22 °C for 5 min. After extracting using chloroform and precipitating with isopropanol, we washed the RNA twice using 75% ethanol, and finally dissolved in RNase-free water. The concentration was determined by NanoDrop2000 spectrophotometer (Thermo, Waltham, MA, USA). Subsequently the RNA was reversely transcribed by transcription kit (RevertAid First Strand cDNA Synthesis. Fermentas) following the instruction. QPCR reactions were performed by UltraSYBR Mixture (CWBIO, Beijing, China), and each sample includes three pair of repetitions. To calculate the relative abundance of mRNA compared with 18 s, we employed the comparative cycle threshold (*C*t) method. Then the RT-PCR and qPCR primer sequences were as follows:

*has-A1CF (-8aa)*/*A1CF* (F: GCCAAGTTTATGATCCCACC; R: CAGTCCTCT CACTCCCGCA);
*hsa-IL-6* (F: GCCACTCACCTCTTCAGAACG; R: CAGTGCCTCTTTGCTG CTTTC).

### 4.4. Protein Extraction and Western Blot Analysis

Proteins were extracted from breast cancer cells with RIPA lysis buffer containing 20 mmol/L (pH 7.5) Tris–HCl, 150 mM NaCl, 1% EDTA, 1% TritonX-100, and 2.5 mM sodium pyrophosphate, supplemented with 1 mM PMSF. Equal amounts of the 30 µg proteins from each extract were separated in 10% SDS-PAGE gel, and then electrophoretically transferred to a PVDF membrane for 50 min in the 300 mA electrical flow. After blocking with 5% free-fat milk in TBST for 1 h at RT, PVDF membranes were incubated with anti-A1CF rabbit pAb (1:1000, ab99955, abcam, Cambridge, MA, USA), anti-IL6 rabbit pAb (1:500, WL01678, Wanleibio, Shenyang, China), anti-PCNA rabbit pAb (1:500, WL01804, Wanleibio, Shenyang, China), and anti-β-Tubulin Mouse mAb (1:2000, HC101-01, Transgen, Beijing, China) overnight at 4 °C, and then incubated with corresponding secondary antibody: horseradish peroxidase-conjugated goat anti-mouse IgG (1:2000, SA00001-1, Proteintech, Wuhan, China) or goat anti-rabbit IgG (1:3000, SA00001-2, Proteintech, Wuhan, China). Finally, the protein bands were visualized with Western blot chemiluminescent HRP substrate reagent (Millipore Corporation, Billerica, MA, USA).

### 4.5. Immunofluorescence

Firstly, MAD-MB-231 cells were grown on the glass coverslips. The cells were fixed with pre-chilled acetone for 15 min, after transiently transfected with *A1CF (-8aa)*, *A1CF*, or null vector for 36 h. Subsequently, cells were, respectively, permeabilized with 1% Triton X-100 (diluted in PBS) for 20 min and blocked with 5% BSA (diluted in PBS) for 1 hour at room temperature. For immunofluorescence staining, the rabbit polyclonal antibody against EGFP (1:1000, ab99955, abcam) and monoclonal anti-flag^®^ M2 antibody produced in mouse (1:1000, F1804, Sigma, Ronkonkoma, NY, USA) were used to probe A1CF overnight at 4 °C, At the meantime, we detected rabbit IgG by the goat anti rabbit IgG-CFL 488 (Santa Cruz Biotechnology, Santa Cruz, CA, USA; 1:2000). In regard to F-actin cytoskeleton staining, MDCK cells were incubated with rhodamine-labeled phalloidin (Sigma; 1:1000) overnight at 4 °C. Finally, nuclei were stained with DAPI (Santa Cruz Biotechnology; 1:5000) for 5 min at room temperature. All of the departments were rinsed with 1× PBS for three times, subsequently, and were softly washed with water. To view under fluorescence microscope, we immobilized the coverslips on the glass slides with 50% glycerol in PBS. At the same time, we took relevant pictures with a SPOT Diagnostic (Sterling Heights, MI, USA) CCD camera. Eventually, immunofluorescent images were performed by using Adobe Photoshop CS3.

### 4.6. 5-Ethynyl-20-Deoxyuridine (EdU) Assays

Simply, breast cancer cells were seeded into 24-well plates. Twelve hours later, cells were, respectively, transfected with 500 ng plasmids of *A1CF (-8aa)*, *A1CF*, and null vector. After 36 h transfection, the proliferation of cells was determined via EdU DNA proliferation and detection kit (RiboBio, Guangzhou, China) based on the manufacturer’s instructions.

### 4.7. Statistical Analysis

All of the experiments were performed in three independent assays, and error bars represented the standard deviation from the mean. Unpaired two-tailed Student’s test was applied to test differences by statistical software GraphPad Prism 5 (GraphPad, Inc., La Jolla, CA, USA), and *p*-values of less than 0.05 were regarded statistically significant. Significant differences were indicated for *p* < 0.05 (*), *p* < 0.01 (**) or *p* < 0.001 (***).

## 5. Conclusions

Taken together, our present study demonstrates, for the first time, that the EIYMNVPV motif is required for A1CF nucleus localization, and A1CF (-8aa) and A1CF are, respectively, located in the cytoplasm and the nucleus. Additionally, based on the detection of EDU and PCNA, A1CF (-8aa) promotes cell proliferation via upregulating the expression of *IL-6* in basal-like breast cancer cells (MAD-MB-231). Therefore, the EIYMNVPV motif or A1CF (-8aa) might be an underlying gene therapeutic target for breast cancer.

## Abbreviations

dsRBM: Double strand RNA binding motif
PCNA: Proliferating cell nuclear antigen
A1CF: Apobec-1 complementation factor
cDNA: Complementary DNA
qPCR: Quantitative reverse-transcription polymerase chain reaction
RRM: RNA recognition motifs
apoB: Apolipoprotein-B
EdU: 5-Ethynyl-20-Deoxyuridine
IL-6: Interleukin-6
